# Examining the functional activity of different obsessive–compulsive symptom dimensions in Tourette syndrome

**DOI:** 10.1016/j.nicl.2020.102198

**Published:** 2020-01-25

**Authors:** Tracy Bhikram, Adrian Crawley, Paul Arnold, Elia Abi-Jaoude, Paul Sandor

**Affiliations:** a Tourette Syndrome Neurodevelopmental Clinic, Toronto Western Hospital, Toronto, Ontario, Canada; b Institute of Medical Science, University of Toronto, Toronto, Ontario, Canada; c Department of Medical Imaging, University of Toronto, Toronto, Ontario, Canada; d Mathison Centre for Mental Health Research & Education, Hotchkiss Brain Institute, Cumming School of Medicine, University of Calgary, Calgary, Alberta, Canada; e Department of Psychiatry, Cumming School of Medicine, University of Calgary, Calgary, Alberta, Canada; f Program in Genetics and Genomic Biology, University of Calgary, Calgary, Alberta, Canada; g Department of Psychiatry, Hospital for Sick Children, Toronto, Ontario, Canada; h Division of Child Psychiatry, Department of Psychiatry, Youthdale Treatment Centers, Toronto, Ontario, Canada

**Keywords:** Tourette syndrome, Obsessive–compulsive symptoms, Symptom provocation, Sensory phenomena, Disgust vulnerabilities, FMRI

## Abstract

**Objectives:**

Tourette syndrome (TS) is commonly comorbid with obsessive–compulsive disorder (OCD) and many phenomenological similarities exist between tics and obsessive–compulsive symptoms (OCS). Therefore, due to the clinical importance of comorbid OCD, the goal of this study was to investigate the neural substrates of OCS in TS using functional magnetic resonance imaging.

**Methods:**

Forty patients with TS and 20 healthy controls underwent functional magnetic resonance imaging while viewing blocks of OCS-provoking pictures relating to washing, checking and symmetry symptoms, as well as generally disgusting and neutral scenes. Statistical comparisons were made between patients with moderate/severe OCS, absent/mild OCS and healthy controls. As well, within the entire TS patient group, significant associations with clinical measures were assessed for each of the provocation conditions.

**Results:**

Group differences in the insula, sensorimotor cortex, supramarginal gyrus and visual processing regions were common among the checking, washing and disgust conditions. In the patient group, negative associations between OCS severity and activity in the supramarginal gyrus, inferior frontal gyrus, sensorimotor cortex, precuneus and visual processing regions were common among the provocation conditions. Tic severity was only associated with activity in the anterior cingulate cortex for the symmetry condition.

**Conclusion:**

Our findings implicate areas previously reported to be involved in OCD, as well as areas not typically implicated in OCD, suggesting that the neurobiological profile of TS+OCD is intermediate to pure TS and pure OCD.

## Highlights

• Patients with Tourette Syndrome completed an obsessive–compulsive provocation task.
• Patients reported higher anxiety for the provocation conditions than did controls.
• Group differences found in the insula, sensorimotor cortex and supramarginal gyri.
• Obsessive–compulsive severity associated with frontal and parietal lobe activity.
• Tic severity associated with anterior cingulate activity for the symmetry condition.

## 1 Introduction

Tourette syndrome (TS) is characterized by the presence of tics, but for most patients with TS, “tics alone are the exception rather than the rule” (Leckman et al., 2006). It is estimated that 86–90% of patients with TS have comorbid psychopathologies, with obsessive–compulsive disorder (OCD) and attention-deficit hyperactivity disorder (ADHD) being the most common comorbid disorders (Groth et al., 2017; Hirschtritt et al., 2015; Freeman et al., 2000). Often these comorbidities are more distressing than the tics and have the greatest impact on quality of life, therefore determining the overall prognosis of TS (Rizzo et al., 2014). OCD in particular can significantly add to the clinical burden of TS, with obsessive–compulsive symptoms (OCS) affecting 50–80% of patients with TS (Hirschtritt et al., 2015; Lebowitz et al., 2012; Goodman et al., 2006; Eddy et al., 2011). Tics and OCS share many phenomenological similarities, both involve involuntary or intrusive repetitive behaviours and often involve sensory phenomena (SP) (Ferrão et al., 2009; Eddy and Cavanna, 2014).

Factor analytic studies have divided the symptom dimensions of OCD into different subtypes including those consisting of forbidden thoughts and checking compulsions, symmetry/ordering symptoms and contamination/cleaning symptoms (Bloch et al., 2008). Previous studies have demonstrated that the OCS profile of patients with TS is significantly different from patients with OCD only. When compared to OCD patients without TS, TS+OCD patients are less likely to have contamination and cleaning symptoms, and are more likely to exhibit symptoms relating to the need for symmetry, violent/sexual images and somatic obsessions, as well as counting, ordering and tic-like compulsions (Holzer et al., 1994; Petter et al., 1998; Diniz et al., 2006). Furthermore, global tic severity has been shown to be associated with the symmetry/ordering and forbidden thoughts/aggression dimensions in children and across all ages, respectively (Kano et al., 2015). As well, tic-like compulsions, which include touching, tapping, rubbing and blinking, differ in nature from other OCS in that they are usually not performed to relieve anxiety, are less frequently preceded by obsessions, and more frequently preceded by SP (de Vries et al., 2016). In fact, it is possible that some compulsions in TS+OCD may actually represent simple or complex tics to which the patient subsequently attaches meaning, so that the obsessions are post hoc constructs to help explain tics (Holzer et al., 1994). Nevertheless, while there do appear to be differences in the content of OCS in TS as compared to pure OCD, many TS patients experience the full range of typical OCS including those related to contamination/cleaning and checking (Holzer et al., 1994).

Further similarities between TS and OCS include exaggerated emotional reactions, with heightened anger and disgust responses common to TS (Budman et al., 2003) and OCD (Bhikram et al., 2017), respectively. Specifically, vulnerabilities to disgust are believed to be key factors in the symptomatology of OCD and have started to shape treatment strategies (Athey et al., 2015; Berle et al., 2012; Melli et al., 2015), but their relationship with TS has not been previously investigated, even though the exacerbating effect of heightened emotions on tics has been well established (Conelea et al., 2008).

The similarities and differences between tics and OCS, and between the different types of OCS in TS, raise questions as to their underlying neurobiology. A key prerequisite for developing and testing theories about the neural correlates of both the elicitation and regulation of OCS is the ability to induce such symptoms in a controlled environment. Symptom provocation paradigms are effective in eliciting neural patterns of activation related to the emergence of OCS (Rotge et al., 2008). In these tasks, OCS are induced by exposing individuals to stimuli that are directly related to concerns typically reported in OCD.

Using provocation tasks, researchers have attempted to discern the neurobiological profiles of the different OCS dimensions in OCD patients. In an early positron emission tomography (PET) study, checking and symmetry symptoms were found to be correlated with increased and decreased cerebral blood flow in the striatum, respectively; whereas washing symptoms correlated with blood flow in the orbitofrontal cortex (OFC) and the anterior cingulate cortex (ACC) (Rauch et al., 1998). Since patients often have more than one type of OCS, Mataix-Cols et al. utilized a dimensional approach to investigate the neural correlates of washing and checking symptoms within the same patients to avoid dividing patients into mutually exclusive subgroups. They found that washing symptoms were correlated with greater activity in the ventrolateral prefrontal cortex, insula, temporal pole and occipital lobe, whereas checking severity was associated with greater activity in the pallidum, putamen, and thalamus. The authors concluded that each symptom dimension might reflect the dysregulation of highly conserved and partially overlapping neural systems that serve to detect, appraise, and respond to potential threats (Mataix-Cols et al., 2004). To consolidate the many OCS provocation neuroimaging findings, Rotge and colleagues performed a meta-analysis of the studies. They found that symptom provocation was associated with activity in numerous cortical and subcortical regions, including the OFC, ACC, dorsolateral prefrontal cortex, precuneus, pallidum, thalamus, hippocampus and superior temporal gyrus (Rotge et al., 2008).

While numerous OCS provocation neuroimaging studies have been conducted in OCD populations, there is a paucity of research on TS+OCD samples. In fact, despite the high comorbidity of OCS in TS, there has only been one OCS provocation neuroimaging investigation conducted in a TS sample. In a PET study, 14 TS patients and 10 controls viewed pictures of symmetrically and asymmetrically ordered objects. During the asymmetric condition, patients reported similar levels of distress as controls, but experienced significantly greater urges to rearrange the objects (de Vries et al., 2013). Within the patient group, increased cerebral blood flow in the ACC, inferior frontal gyrus (IFG) and supplementary motor area (SMA) was observed during the asymmetrical condition as compared to baseline. When compared to controls, TS patients showed significantly larger increases of cerebral blood flow in the ACC and smaller increases in the occipital cortex, motor cortex and dorsal-medial prefrontal cortex (de Vries et al., 2013). While novel, this study had important limitations, as symmetry was the only OCS dimension investigated. As well, only 2 of the patients had comorbid OCD, so it is not clear to what extent the paradigm was actually provoking OCS.

Considering how common and debilitating OCS can be in TS, the goal of the current study was to determine the neural substrates of the different OCS dimensions in a sample of TS patients using a validated provocation paradigm and functional magnetic resonance imaging (fMRI).

## 2 Methods

### 2.1 Participants

A total of 40 adult patients with TS were recruited from the Tourette Syndrome Neurodevelopmental Clinic at the Toronto Western Hospital in Toronto, Canada. A further 20 age, sex and education-matched healthy controls, without a personal history of psychiatric illness as determined by the Mini International Neuropsychiatric Interview, were also recruited from the community. All participants had to have been right-handed and between the ages of 18 and 65. Conversely, participants were not eligible to participate in the study if they had a head injury or history of seizures, were pregnant at the time of the study, had a history of substance abuse or dependence within the last 6 months or had any contraindications to the MRI environment. For the patient group, further exclusion criteria included a diagnosis of a comorbid autism spectrum disorder or any psychotic disorder. All study procedures were approved by the University Health Network's research ethics board and conducted in compliance with the Declaration of Helsinki. Written informed consent was obtained from all participants prior to the commencement of study procedures.

### 2.2 Instruments

The following instruments were used to assess various clinical and behavioural measures:

• *Yale Global Tic Severity Scale (YGTSS)* is a semi-structured, clinician-rated instrument that is considered to be the gold standard when assessing tic severity (Leckman et al., 1989). The number, frequency, intensity, interference and complexity of motor and phonic tics within the previous week were assessed to produce a ‘Total Tic Score’ subscale, rated from 0–50.
• The self-report version of the *Yale-Brown Obsessive–Compulsive Scale (Y-BOCS)* was used to measure OCS severity (Baer et al., 1993). Specifically, the severity of obsessions and compulsions were measured with 10 questions pertaining to the time occupied by obsessions/compulsions, interference caused by OCS, distress, resistance and degree of control over OCS.
• The *Obsessive–Compulsive Inventory* (*OCI-R*) is also a self-report questionnaire that assesses the severity of OCS (Foa et al., 2002). However, unlike the Y-BOCS, the OCI contains subscales that quantifies the severity of different OCD symptom dimensions (e.g. contamination/washing, checking, symmetry/ordering, etc.).
• The *Disgust Sensitivity and Propensity Scale* (*DPSS-R*) is a self-report scale that measures disgust propensity (i.e. the frequency and/or intensity in which one generally responds with disgust) and disgust sensitivity (i.e. the degree of negativity associated with the elicitation and experience of disgust), irrespective of specific elicitors (van Overveld et al., 2010).
• The *Disgust Scale* (*DS-R*) is also a self-report scale that measures the degree to which different types of stimuli elicit disgust responses (Olatunji et al., 2007). Specifically, the DS-R measures sensitivities to different types of disgust (i.e. core disgust, animal-reminder disgust and contamination disgust).
• The English version of the *University of Sao Paulo Sensory Phenomena Scale* (*USP-SPS*) is a semi-structured scale developed to investigate the presence and severity of different types of SP that precede repetitive behaviours such as tics and OCS, by probing the frequency, amount of distress and the degree of interference caused by SP (Rosario et al., 2009).
• The *Adult ADHD Self-Report Scale (ASRS)* is a self-report screener for ADHD and comprises 18 questions relating to the DSM-IV-TR ADHD criteria and specifically pertains to adults (Kessler et al., 2005). If answers to any 4 of the 6 questions in part A met a certain threshold, the participant was deemed to have symptoms highly consistent with a diagnosis of ADHD.

### 2.3 Symptom provocation paradigm

All participants completed three 7-minute runs in which they viewed blocks of symptom-related, neutral and generally aversive scenes taken from the Maudsley Obsessive–Compulsive Stimuli Set (MOCSS). The MOCSS is a standardized stimulus set depicting common OCD triggers corresponding to the OCS dimensions of contamination, checking and symmetry (Mataix-Cols et al., 2009). As well, neutral and generally aversive (i.e. disgusting) pictures selected from the International Affective Picture System are also included as part of the MOCSS (Lang et al., 1999). The MOCSS has good validity and can reliably provoke OCS in patients with OCD and in healthy volunteers (Mataix-Cols et al., 2009; Mataix-Cols et al., 2003).

For the current study, each run was composed of 10 blocks pertaining to the above-mentioned categories, with each block displaying 8 pictures from a particular picture category for 2 s each. Blocks were displayed in a pseudo-randomized order so that there were never 2 consecutive blocks of the same category. Examples of pictures from the washing category included those displaying money, a syringe and elevator buttons; those from the checking category included plugged in electric appliances, an open door, and a lit stove; and those from the symmetry category included uneven/disorderly environments. Pictures relating to the neutral category included nature scenes and furniture, whereas the disgusting scenes included images of insects, decaying foods and dirty toilets. Efforts were made to ensure that the pictures used in the washing blocks would not be perceived as very disgusting by healthy volunteers. In total, 48 pictures were used for each of the categories with no repetition of any stimulus.

Each block began with 8-second instructions displayed on a screen and viewed via MRI compatible goggles. Instructions were tailored for each of the block categories and informed the participant how to imagine themselves in relation to the stimuli. At the end of each picture block, participants were asked to rate how anxious the pictures made them feel using a visual analog scale that they could see on the screen and respond to via a button box. The visual analog scale contained nine numerical anchor points ranging from 0 to 8 with the two extremes further defined with text (‘no anxiety’ and ‘extremely anxious’, respectively). Prior to the scan, all subjects underwent a training procedure using only neutral stimuli to familiarize themselves with the task and instructions. The pictures used for the training were different from the ones shown during the scan. Similar study designs have frequently been conducted in OCD populations, with the MOCSS being a stimuli set that is commonly used in fMRI settings (Mataix-Cols et al., 2004; Gilbert et al., 2009; Agarwal et al., 2013; An et al., 2009; Schienle et al., 2005).

### 2.4 Image acquisition

Images were acquired with a 3.0-T GE clinical scanner (GE Medical Systems, Milwaukee, Wisc.) using an 8-channel head coil at Toronto Western Hospital. T1-weighted image parameters were as follows: echo time = 2.7 ms, inversion time = 450 ms, flip angle = 15°, slice thickness = 1.0 mm, number of slices = 176, field of view = 22 × 22 cm^2^, and matrix size = 220 × 220. T2*-weighted image parameters were as follows: repetition time = 2.4 s, echo time = 30 ms, flip angle = 70°, slice thickness = 3.5 mm, number of slices = 41, field of view = 22.4 × 22.4 cm^2^, and matrix size = 64 × 64.

## 3 Data analysis

### 3.1 Clinical and behavioural data

Two-sample t-tests were used to determine significant differences between the patient and control groups for scores on the Y-BOCS, OCI, USP-SPS, DS-R and the DPSS-R. Subjective anxiety ratings for each of the blocks were grouped by category and then averaged. Two-sample *t*-tests were then conducted to detect group differences in subjective anxiety ratings for each of the categories. Within the patient group, a series of multiple regression analyses were conducted between each of the subjective anxiety ratings and measures of the Y-BOCS, DPSS – sensitivity subscale, the USP-SPS and the YGTSS – total tic score. Scores from the OCI and DS-R were excluded from the regression analyses due to collinearity with either Y-BOCS and/or DPSS-R scores. All tests were considered significant at *p* < 0.05 after correction for multiple comparisons.

### 3.2 Imaging data

All fMRI data preprocessing and analysis steps were conducted using SPM 12 (Wellcome Department of Imaging Neuroscience, London [http://www.fil.ion.ucl.ac.uk/spm]). The first three images from each run were excluded from the analyses to eliminate any T1-equilibrium effects. Images underwent standard preprocessing that included motion correction, segmentation using template tissue probability maps for gray and white matter and cerebrospinal fluid (International Consortium for Brain Mapping), normalization to the Montreal Neurological Institute (MNI) EPI template, and spatial smoothing with a Gaussian filter set at 8 mm full width at half-maximum. Statistical parametric analyses were then carried out to obtain general linear model contrasts between the “provocation” and the “neutral” blocks for each of the provocation conditions (i.e. checking, washing, symmetry and disgust), for each participant.

ANOVAs comparing patients with moderate/severe OCS (defined by Y-BOCS score ≥ 16; *n* = 19) to patients with mild/absent OCS (Y-BOCS score <16; *n* = 21) and healthy controls were conducted for each of the provocation conditions. This cut-off score is based on the scoring categorizations of the Y-BOCS (Tolin et al., 2005). Additionally, within the patient group, regressions were performed for each of the provocation conditions. Specifically, subjective anxiety ratings, YGTSS Tic Totals, Y-BOCS, USP-SPS and DPSS sensitivity subscale scores were used as covariates of interest [to be consistent with the variables used in the behavioural regression analyses above], while controlling for ADHD presence and any psychotropic medication usage, including anti-tic and anti-obsessional medications. Tests were considered significant at *p* < 0.001 (uncorrected) and then FWE corrected for multiple comparisons (*p* < 0.05) at the cluster level.

## 4 Results

### 4.1 Behavioural measures

**Fig. 1:**
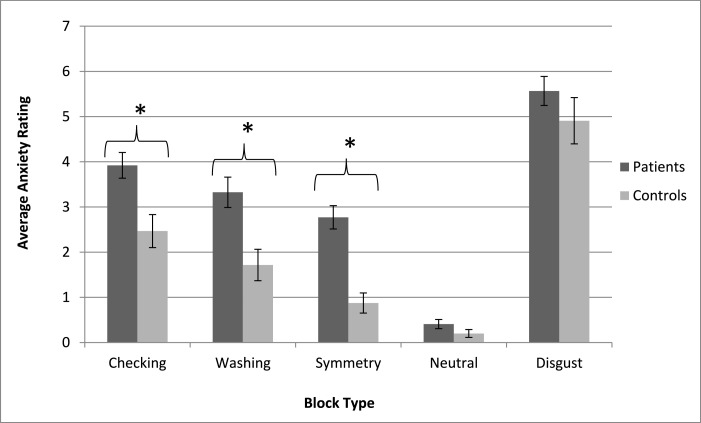
Subjective anxiety ratings for each of the block conditions. *Differences significant at *p* < 0.05 corrected for multiple comparisons.

With the exception of scores on the DS-R, the disgust propensity subscale of the DPSS, and the contamination/washing subscale of the OCI, patients had significantly higher scores for all of the clinical and behavioural instruments when compared to the control group (Table 1). Additionally, patients reported significantly higher levels of subjective anxiety for each of the symptom related provocation conditions of the task (i.e. checking, symmetry, washing) than the controls did, but no such significant differences existed for either the neutral or disgust conditions (Fig. 1). Finally, the multiple regression models used to predict subjective anxiety scores from scores on the Y-BOCS, YGTSS, USP-SPS and DPSS were only significant for the checking and symmetry conditions (Table 2). Specifically, Y-BOCS scores significantly predicted checking (F(4,35) = 3.99; *p* = 0.01; r^2^ = 0.33) and symmetry-related anxiety ratings (F(4,35) = 3.64; *p* = 0.015, r^2^ = 0.31). When corrected for multiple comparisons, no variables predicted ratings for the washing, disgust and neutral blocks; as such, the overall models were not significant. Results from additional behavioural analyses are detailed in Supplementary Tables 1–4.

**Table 1:** Comparisons between patients and controls for various clinical/behavioral measures. *Results significant at *p* < 0.05 corrected for multiple comparisons.

	Patients	Controls	
	Mean (SD)	Mean (SD)	
Age, years	37.1 (13.1)	36.9 (17.2)	*t* = 0.03
Education, years	16.5 (1.80)	16.9 (2.83)	*t* = 0.71
YGTSS Total Tic Score	25.6 (8.95)	N/A	N/A
Y-BOCS	13.0 (7.30)	1.32 (2.50)	*t* = 9.10*
OCI total	24.1 (15.3)	3.65 (6.90)	*t* = 7.15*
OCI – checking	4.24 (3.52)	0.91 (1.58)	*t* = 4.48*
OCI – contamination	3.18 (3.23)	1.09 (2.21)	*t* = 2.01*
OCI – ordering	5.47 (3.15)	1.45 (2.34)	*t* = 3.92*
USP-SPS	7.89 (4.08)	0.30 (1.13)	*t* = 10.6*
DPSS – propensity	15.8 (4.40)	14.5 (3.30)	*t* = 1.45
DPSS – sensitivity	12.5 (3.90)	8.50 (1.90)	*t* = 4.84*
DS-R	1.92 (0.69)	1.65 (0.42)	*t* = 1.48
	Patients (n)	Controls (n)	
Gender, Male:Female	31:8	16:4	χ^2^=0
OCD diagnosis	20	0	N/A
Likely ADHD	16	0	N/A
Psychotropic medication usage	25	0	N/A
α−2 adrenergic agonists	4	0	N/A
Antipsychotics	1	0	N/A
SSRIs/anxiolytics	18	0	N/A
Stimulants/norepinephrine reuptake inhibitors	6	0	N/A

**Table 2:** Regression table of severity scores and their relationship with the average subjective anxiety ratings for each of the picture conditions in the patient group. Numbers represent the t score for the regression and are significant at *p* < 0.05 corrected for multiple comparisons*.

	Checking	Symmetry	Washing	Neutral	Disgust
Y-BOCS	3.33*	3.52*	2.22	1.52	2.31
YGTSS Tic Score	0.69	0.213	0.230	0.371	−0.773
USP-SPS	0.67	0.415	0.49	−0.023	0.242
DPSS – sensitivity	−2.37	−1.86	−0.53	−1.131	−1.252

### 4.2 Imaging measures

#### 4.2.1 Group comparisons

**Fig. 2:**
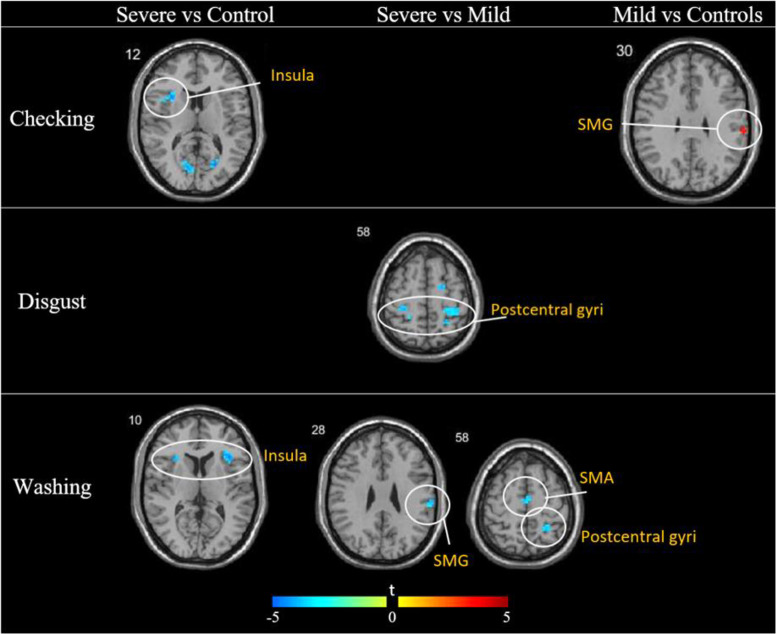
Comparisons between patients with moderate/severe OCS, absent/mild OCS and healthy controls for each of the provocation conditions. There were no significant differences between the groups for the symmetry condition.

For the checking contrasts, controls exhibited greater activity than the severe patient group in the insula and the calcarine sulcus, whereas the mild patient group exhibited greater activity in the supramarginal gyrus when compared to controls. For the disgust contrasts, the only significant differences were between the severe and mild patients; mild patients exhibited greater activity in the superior frontal gyrus, calcarine sulcus, and bilaterally in the postcentral gyri. For the washing contrasts, severe patients exhibited less activity than controls bilaterally in the insula, and less activity than mild patients in the SMA, postcentral and supramarginal gyri (Fig. 2, Table 3). There were no significant differences between any of the groups for the symmetry contrasts.

**Table 3:** Group comparisons between TS patients with moderate/severe OCS, absent/mild OCS and healthy controls for the checking, disgust and washing conditions.

Condition	Group Comparison	Region	BA	MNI coordinates			Peak z score	k
Checking	Controls > Severe	Calcarine	18	21	−70	13	3.92	110
		Insula	48	−33	17	13	3.85	105
		Calcarine	17	−15	−73	10	3.69	95
	Mild > Controls	Supramarginal	48	60	−25	31	3.60	74
Disgust	Mild > Severe	Calcarine	19	−24	−67	7	4.01	85
		Postcentral	3	−30	−31	55	3.76	101
		Sup. Frontal	6	18	−6	61	3.67	76
		Postcentral	4	36	−31	61	3.50	109
Washing	Controls > Severe	Insula	48	35	23	10	3.92	80
		Insula	48	−33	23	10	3.72	88
	Mild > Severe	Supramarginal	48	63	−22	28	3.60	74
		Postcentral	2	24	−49	58	3.50	79
		SMA	6	−3	−10	58	3.47	76

#### 4.2.2 Within-patient regressions

Within the entire patient group, greater Y-BOCS scores were associated with less activity in the IFG, precentral gyrus, superior temporal gyrus, supramarginal gyrus and in visual processing areas during the checking blocks. Similarly, for the disgust blocks, greater Y-BOCS scores were associated with less activity in the inferior and middle frontal gyri, inferior and superior parietal lobules, angular gyrus, supramarginal gyrus, precentral gyrus, postcentral gyrus, SMA, superior and middle temporal gyri and visual processing areas. For the symmetry blocks, Y-BOCS scores were negatively associated with activity in the precentral gyrus, SMA, insula, midcingulate, IFG, supramarginal gyrus and the calcarine sulcus. Finally, negative associations were found between Y-BOCS scores and activity in the supramarginal gyrus, precuneus and calcarine sulcus for the washing condition (Table 4, Supplementary Figure 1).

**Table 4:** Within-patient group associations with clinical/behavioural measures for each of the provocation conditions.

Condition	Region	BA	MNI coordinates			Peak z score	k
*Covariate: Y-BOCS (negative associations)*							
Checking	Inferior Frontal	48	36	26	28	4.15	112
	Middle Occipital	39	−36	−73	25	3.95	104
	Calcarine	17	−3	−79	10	3.90	158
	Precentral	6	−33	−10	46	3.60	77
	Superior Temporal	22	57	−22	4	3.58	108
	Supramarginal	2	66	−22	28	3.50	75
	Precuneus	7	−9	−70	37	3.40	85

Disgust	Middle Frontal	46	33	20	40	4.53	90
		44	−39	26	34	4.26	203
	Superior Parietal	7	15	−64	52	4.27	215
	Inferior Frontal	44	57	11	25	4.12	123
	Supramarginal	40	51	−37	43	4.12	187
		40	−63	−40	34	3.84	85
	Precentral	6	−35	−10	40	4.11	158
		6	39	2	52	3.50	75
	Postcentral	2	−21	−43	67	3.60	74
		3	39	−34	58	3.51	79
	SMA	8	−6	23	49	3.94	89
	Inferior Parietal	3	−51	−19	37	3.97	100
	Middle Occipital	39	−36	−73	19	3.91	102
	Precuneus	7	−9	−70	40	3.52	129
	Superior Temporal	48	51	−25	10	3.51	116
	Middle Temporal	37	42	−61	7	3.75	111
	Calcarine	18	6	−70	19	3.65	125

Symmetry	Precentral	6	−33	−7	43	4.20	105
		6	−54	2	43	3.65	78
	SMA	8	−3	26	49	3.94	187
	Inferior Frontal	48	−39	8	22	3.93	81
	Insula	48	−33	23	10	3.76	112
	Midcingulate	24	0	17	40	3.69	87
	Calcarine	17	−6	−76	16	3.65	212
	Supramarginal	40	−62	−36	37	3.47	90
		48	54	−34	28	3.43	180

Washing	Supramarginal	2	66	−25	28	3.73	85
	Precuneus	7	−12	−70	37	3.58	92
	Calcarine	17	−12	−79	10	3.58	115

*Covariate: YGTSS (positive associations)*							

Symmetry	ACC		−7	26	19	3.68	90

*Covariate: DPSS Sensitivity Subscale (negative associations)*							

Disgust	Vermis		−3	−46	4	3.64	95
	Superior Temporal	21	54	2	−14	3.61	150
	ACC	32	−9	35	19	3.53	103
	Putamen		35	−9	2	3.50	112

*Covariate: USP-SPS (negative associations)*							

Disgust	Superior Temporal	48	60	−7	−2	3.91	109
	Precentral	6	−42	−1	37	3.50	74
	Middle Frontal	46	−29	17	40	3.50	76
	Rolandic Opercula	48	−57	−1	4	3.45	114

Associations with total tic scores from the YGTSS were only significant for the symmetry blocks; higher severity scores were associated with greater activity in the ACC. Associations between brain activity and scores on the DPSS and USP-SPS were only significant for the disgust blocks. Greater disgust sensitivity was associated with less activity in the superior temporal gyrus, ACC, putamen and vermis, whereas negative associations with USP-SPS were found in the superior temporal gyrus, precentral gyrus, middle frontal gyrus and insula. There were no significant associations with subjective anxiety ratings for any of the provocation conditions (Table 4, Supplementary Figure 2).

## 5 Discussion

To the best of our knowledge, this is the first OCS provocation study to be conducted in a TS sample using stimuli from multiple symptom dimensions. The paradigm, while validated in OCD samples, appears to also be effective at provoking OCS in TS, as patients reported significantly greater levels of subjective anxiety than controls for each of the symptom-related provocation conditions, and not for the neutral or general disgust conditions. Moreover, in the patient group, OCS severity scores significantly predicted subjective anxiety ratings for the checking and symmetry-related conditions, indicating that the paradigm was indeed tapping into OCS related anxiety rather than general anxiety.

Within the TS group, negative associations between OCS severity and activity in the supramarginal gyrus, precuneus and visual processing regions were common to all of the provocation conditions. Moreover, with the exception of the washing condition, negative associations between OCS severity and activity in the precentral gyrus, SMA and IFG were also found in all of the provocation conditions. Group differences between severe patients, mild patients and controls involved many of these same regions including the supramarginal gyrus and the SMA, as well as the insula and postcentral gyrus. The functions of these regions and their putative role in TS and OCD will be discussed below.

The parietal lobe, especially the supramarginal gurus and precuneus, has been a recent focus of research in OCD and TS populations (Eddy, 2016). The supramarginal gyrus is part of the somatosensory association cortex and is important for the neural representation of motor actions; lesions in this area have been associated with deficits in the generation of mental movement representations (Sirigu et al., 1996). This relates to the symptomatology of both OCD and TS, as mental movement representations play an important role when inhibiting tics and when deciding whether a compulsion should be performed, and if it has been executed sufficiently. The precuneus is involved in self-generated thoughts and the reallocation of attentional resources which might relate to patients’ efforts to distract themselves from obsessive and distressing thoughts triggered by the provocation task (Cavanna and Trimble, 2006). Numerous studies have reported on their involvement in the pathophysiology of OCD and their possible function as endophenotypes associated with increased OCD risk (Menzies et al., 2008; Peng et al., 2014) and OCS severity (Koprivova et al., 2009; Szeszko et al., 2005); therefore the role of the supramarginal gyrus and precuneus in OCS is likely not specific to OCS in TS+OCD, but OCD in general.

The IFG has an important role in response inhibition and its activity has been found to be attenuated in OCD patients (Roth et al., 2007) which is supported by the results of this study, indicating further similarities between TS+OCS and traditional OCD. A meta-analysis of OCS provocation studies found that multiple clusters in the IFG had significant likelihoods of activation, highlighting the importance of the IFG in the pathophysiology of OCD (Rotge et al., 2008). In addition to being implicated in OCD, the IFG is also thought to be involved in TS, with studies reporting significant IFG activity at tic onset (Bohlhalter, 2006; Stern et al., 2000), and during tic (Tinaz et al., 2014) and blink suppression (Mazzone et al., 2010), with activity correlating with tic severity and suppression ability. Along with being responsible for the control of impulsive motor responses, the IFG is also involved in the inhibition of distracting emotional stimuli, which together, allows for the active inhibition of motor and attentional processes across sensory modalities (Corbetta and Shulman, 2002; Mitchell et al., 2008). Therefore, differential activity in this area in TS could reflect efforts to control emotional or motor responses to the provoking OCS stimuli, and more generally, could reflect impairments in the ability to distract oneself from urges to tic and perform compulsions during voluntary suppression.

Unlike the previously discussed regions, abnormal activity in sensorimotor processing regions, including the SMA, precentral and postcentral gyri, are less commonly reported in OCD populations during symptom provocation, demonstrating potential neurobiological distinctions between OCS in TS and traditional OCD. Sensorimotor regions are, however, consistently implicated in the etiology of TS (Wang et al., 2011; Hampson et al., 2009; Ganos et al., 2014; Neuner et al., 2014). Electrical stimulation of the somatosensory cortex evokes somatic sensory experiences, whereas SMA stimulation produces urges to move, unusual sensations, and in some cases, tic-like movements (Fried et al., 1991). Additionally, the SMA was an effective target for treatment in an open-label trial with low frequency transcranial magnetic stimulation, producing symptom improvement in both TS and OCD patients (Mantovani et al., 2006). The observed activation abnormalities of the sensorimotor cortex during the OCS provocation paradigm in the current study is a further reflection of the close phenomenological relationship between TS and OCD.

During the washing and checking conditions, patients with moderate/severe OCS exhibited significantly less activity than controls in the insula, a region commonly reported to have aberrant activity in TS during tic suppression (Tinaz et al., 2015) and tic onset (Bohlhalter, 2006; Neuner et al., 2014) and in OCD patients during symptom provocation (Gilbert et al., 2009; Schienle et al., 2005; Nakao et al., 2005). The insula is believed to be a cortical site for integrated interoception where information about all bodily sensations, including emotional reactions, cognitions, and motor responses, converge (Lerner et al., 2009). It was recently proposed that the insula serves as a nexus linking the sensory and emotional features of premonitory urges with their translation into tics (Conceicao et al., 2017), and thus may also function similarly in OCD, prompting compulsion performance. The observed findings of reduced insular activity in patients with moderate/severe OCS may reflect impairments in the ability to integrate body-state information with emotional signals and cognitive/motor plans, possibly resulting in the increased salience of urges and obsessions. Alternatively, due to the insula's role in representing the physical state of the body, such impairments may affect its ability to evaluate urge-associated actions to generate a sense of relief after tic/compulsion performance.

Tic severity was only associated with activity in the symmetry condition, an interesting finding since symmetry/ordering-related symptoms are one of the most common OCS subtypes in TS populations (Holzer et al., 1994; Petter et al., 1998). This finding adds to existing evidence that symmetry-related OCD is closely related to TS and the tic-subtype of OCD (de Vries et al., 2016). Specifically, tic severity was associated with greater activity in the ACC, a region believed to be involved in the pathophysiology of both OCD and TS (Ganos et al., 2013; Del Casale et al., 2011). Dysfunctional processing in the ACC has been hypothesized to result in error monitoring deficits, generating excessive error signals that are manifested as obsessions and SP, which in turn prompts corrective actions such as tics and compulsions (Schwartz, 1998). In OCD, faulty error processing results in error detection even when no such errors occur and is not silenced even when the desired result has been achieved, explaining symptoms like constant doubt and the need for repetition (Del Casale et al., 2011). As well, the ACC has a role in the regulation of movement, specifically in transitioning premotor functions to behavioural states (Devinsky et al., 1995). Its role in premotor functions, coupled with its ability to produce sensory symptoms as detected by electrical stimulation, may explain its role in TS. Here, the ACC may participate in the motivational aspect of tic performance and suppression by providing inhibitory control mechanisms to regulate tic behaviour (Lerner et al., 2009; Brown et al., 2018).

The inclusion of the disgust condition allowed for the exploration of neural regions related to general emotional reactivity, independent of OCS symptoms. Disgust was the only condition significantly associated with SP scores; similarly, the most robust and widespread associations with OCS severity occurred in the disgust condition. Interestingly, disgust sensitivity scores, which measure the degree of negativity associated with the experience of disgust, were elevated in the patient group as compared to the control group. However, there were no significant differences between the two groups in the triggers that provoke disgust responses, in the subjective anxiety reported during the disgust blocks, or in disgust propensity scores, which measures the frequency of disgust responses. This suggests that patients are not more easily disgusted than healthy controls, but they perceive the feeling of disgust as being more negative and troublesome. The relationship between disgust and SP is not immediately apparent, but they can both involve strong somatosensory and visceral sensations and are perhaps more strongly linked in TS+OCD patients. It is also possible that the significant differences in insula activity detected between patients with moderate/severe OCS and healthy controls during the washing condition may have in part reflected differences in disgust sensitivities since there were no significant differences between the groups for scores on the OCI-washing subscale and insular activity has a well-established relationship with disgust responses (Chapman and Anderson, 2012). Indeed, washing-related OCS are less frequently reported in TS+OCS populations as compared to pure OCD, and was the least common type of OCS reported in the present TS sample (Supplementary Table 1).

Interestingly, we detected no significant clusters of activation in the OFC or the caudate, two of the most consistently implicated regions in OCD (Rotge et al., 2008; Whiteside et al., 2004). In fact, dysfunctions in the orbitofrontal loop of the cortico-striato-thalamo-cortical circuit are hypothesized to contribute to the etiology of OCD, although our findings suggest this may not be the case for OCD in TS. Specifically, the lack of findings in the OFC and caudate, coupled with the observed activity in the sensorimotor cortex that is more typical of TS than OCD, suggests that OCS in the context of TS may be neurobiologically different from pure OCD. The OFC and caudate are important in affective processing, playing a role in the cycle that reinforces obsessions and compulsions and their accompanying anxiety (Menzies et al., 2008). However, the symptomatology of OCS in TS does not always involve the obsessions and anxiety that are typical of OCD; instead they often involve somatosensory or visceral sensations that perhaps do not involve the orbitofrontal circuit, but could potentially reflect the involvement of the sensorimotor cortex. For example, engaging in symmetry and counting-related compulsions may be more likely to be associated with achieving a visceral sense of completeness or relief of unpleasant sensations rather than ameliorating anxiety or averting a dreaded consequence (Kano et al., 2015; Ferrão et al., 2012). Abnormal activation of regions found to be related to both TS and OCD, such as the ACC and IFG, further demonstrates the relatedness of TS and OCD and provides possible explanations as to why OCS are so common in TS. Perhaps faulty activity in such regions produce both tics and OCS, with the full symptom profile of any one patient ultimately dependent on the other regions that make up the aberrant network.

While novel, this study was not without limitations. We did not include a sample of patients with ‘pure’ OCD which would have allowed for direct comparisons between OCS in TS and OCS without any associated tics. Secondly, though stimuli from the most predominant OCD symptom dimensions were used in this study, we did not use stimuli relating to other OCS subtypes that are more common in TS than pure OCD, such as compulsions relating to counting, tapping, touching and other tic-like compulsions. Investigating these other types of OCS will likely further illuminate the differences and similarities between OCS and tics in TS. Thirdly, subjective anxiety ratings recorded during the task were not significantly associated with brain activity for any of the provocation conditions. While this could be because OCS in TS are not always associated with anxiety, it could potentially also be the result of using stimuli that were not provoking or relevant enough even though reported patient anxiety was significantly greater than controls. Standardized stimuli can be less effective at provoking anxiety when compared to idiosyncratic stimuli, however they are more practical for MRI settings and allow for generalizability (De Putter et al., 2017). As well, for the group comparisons, we opted to use Y-BOCS scores to differentiate patients with absent/mild OCS from those with moderate/severe OCS. While the cut-off score of 16 was chosen based on Y-BOCS scoring categorizations, such scores do not always map onto diagnoses, and is therefore a limitation. However, this method was still preferred because it is based on the current severity of OCS, which in turn, may better capture the true clinical picture of the patients, and is a common method used in the literature (Tinaz et al., 2014; Debes et al., 2011; Pourfar et al., 2011). Additionally, while the presence of ADHD was controlled for, ADHD severity was not measured, therefore more subtle associations with ADHD could not be assessed or controlled. Finally, while medications were controlled for in the regression analyses, they were not controlled for in the group comparisons, nor were the individual effects of the different classes of medications considered. Based on previous research in OCD populations, medications frequently have a ‘normalizing’ effect on regional abnormalities (Nakao et al., 2014), which could have potentially obfuscated more subtle differences between TS patients and controls, but also reinforces the involvement of the regions that were found to be significant.

This is, to our knowledge, the first report on the neural correlates of checking, washing and symmetry-related OCS in a sample of TS patients. Our findings implicate the involvement of areas previously reported to be involved in OCD, including the IFG, parietal lobule, and insula, as well as areas not typically implicated in OCD, such as the sensorimotor cortex. This suggests that TS+OCD shares neuropathology with both TS and OCD. While it is possible that “pure” TS and “pure” OCD fall on different ends of a neurobiological spectrum, with TS+OCD intermediate to both disorders as some investigators have previously hypothesized (Diniz et al., 2006; Cath et al., 2001), it is more likely that their relationship is more complicated. Perhaps, TS+OCD share partially overlapping circuitries with both TS and OCD, with symptomatology, SP and associations with other comorbidities serving as modulators of their relationship. However, more research is needed to further elucidate the neurobiological relationship between TS and OCD, and their associations with related clinical measures.

## Funding

This work was supported by the Wolf Family Chair in Neurodevelopmental Psychiatry, Toronto General & Western Hospital Foundation. The funders had no role in the study design, data collection and analysis, decision to publish, or in the preparation of this manuscript.

## CRediT authorship contribution statement

**Tracy Bhikram:** Conceptualization, Investigation, Formal analysis, Writing - original draft. **Adrian Crawley:** Conceptualization, Formal analysis, Writing - review & editing. **Paul Arnold:** Conceptualization, Formal analysis, Writing - review & editing. **Elia Abi-Jaoude:** Conceptualization, Formal analysis, Writing - review & editing. **Paul Sandor:** Conceptualization, Resources, Formal analysis, Writing - review & editing.

## Declaration of Competing Interest

Dr. Arnold receives support from the Alberta Innovates Translational Health Chair in Child and Youth Mental Health. Dr. Sandor receives grant funding from the Tourette Syndrome Association for an unrelated study. All other authors have no declarations of interest.

## Appendix Supplementary materials

Image, application 1
