# Supplementary material for: Examining the functional activity of different obsessive–compulsive symptom dimensions in Tourette syndrome
Source: Neuroimage Clin. 2020 Jan 25;26:102198. doi: 10.1016/j.nicl.2020.102198 (PMC7025096; doi:10.1016/j.nicl.2020.102198)
Supplement: Supplementary file 1 [file mmc1.docx]

**Examining the Functional Activity of Different Obsessive-Compulsive Symptom Dimensions in Tourette Syndrome**

Supplementary Table 1. Percentage of patients with symptoms from different OCS dimensions

|  | | Patients (%) |
| --- | --- | --- |
| Obsessions | Aggressive | 60 |
|  | Symmetry | 55 |
|  | Contamination | 43 |
| Compulsions | Checking | 53 |
|  | Ordering | 40 |
|  | Cleaning | 30 |

Supplementary Table 2. Clinical and behavioural measures between TS patients with moderate/severe OCS, mild/absent OCS and healthy controls

|  | **Patients with Y-BOCS ≥ 16**  *n = 19*  Mean (SD) | **Patients with Y-BOCS < 16**  *n = 21*  Mean (SD) | **Controls**  *n = 20*  Mean (SD) | **Statistics** |
| --- | --- | --- | --- | --- |
| **Y-BOCS** | 19.0 (2.22) | 6.53 (5.44) | 1.32 (2.50) | F=102; p<0.001; a,b,c |
| **OCI total** | 32.3 (9.95) | 14.3 (12.0) | 3.65 (6.90) | F= 32.5; p<0.001; a,b,c |
| **OCI – checking** | 6.06 (3.11) | 2.33 (3.11) | 0.91 (1.58) | F=10.4; p<0.001; a,c |
| **OCI – contamination** | 3.63 (3.30) | 1.93 (2.79) | 1.09 (2.21) | F=2.81; p=0.071 |
| **OCI – ordering** | 7.00 (3.29) | 3.87 (2.42) | 1.45 (2.34) | F=10.9; p<0.001; a,b |
| **USP-SPS** | 8.20 (4.56) | 7.31 (4.11) | 0.30 (1.13) | F=33.3; p<0.001; a,b |
| **DPSS – propensity** | 16.9 (4.60) | 14.3 (4.25) | 14.5 (3.30) | F=2.83; p=0.068 |
| **DPSS – sensitivity** | 13.2 (4.59) | 9.93 (2.66) | 8.50 (1.90) | F=11.3; p<0.001; a |
| **DS-R – animal reminder** | 2.02 (0.80) | 1.75 (0.50) | 1.66 (0.85) | F=2.25; p=0.117 |
| **DS-R – contamination** | 1.65 (1.04) | 1.33 0.82) | 1.28 (0.54) | F=1.68; p=0.198 |
| **DS-R – core**  Note: significant post-hoc differences are signified by  a = severe patients vs. controls  b = mild patients vs. controls  c = severe patients vs. mild patients | 2.18 (0.76) | 1.98 (0.59) | 1.80 (0.39) | F=2.99; p=0.060 |

Supplementary Table 3. Subjective anxiety ratings between TS patients with moderate/severe OCS, mild/absent OCS and healthy controls for each of the block conditions

| **Block Condition** | **Patients with Y-BOCS ≥ 16**  *n = 19*  Mean (SD) | **Patients with Y-BOCS < 16**  *n = 21*  Mean (SD) | **Controls**  *n = 20*  Mean (SD) | **Statistics** | | |
| --- | --- | --- | --- | --- | --- | --- |
|  |  |  |  | **F-test** | **p value** | **Post hoc** |
| **Checking** | 4.50 (1.4) | 3.34 (1.8) | 2.47 (1.6) | 8.37 | 0.001 | a, c |
| **Washing** | 3.53 (2.1) | 2.96 (2.1) | 1.72 (1.6) | 5.34 | 0.007 | a |
| **Symmetry** | 3.46 (1.6) | 2.12 (1.3) | 0.88 (1.0) | 18.2 | <0.001 | a, b, c |
| **Neutral** | 0.55 (0.7) | 0.34 (0.7) | 0.20 (0.4) | 2.19 | 0.121 |  |
| **Disgust** | 5.89 (2.0) | 5.24 (1.9) | 4.91 (2.3) | 1.48 | 0.236 |  |

Note: significant post-hoc differences are signified by

a = severe patients vs. controls

b = mild patients vs. controls

c = severe patients vs. mild patients

Supplementary Table 4. Within-patient correlation coefficients between OCI-R subscale scores and the corresponding dimensional subjective anxiety ratings from the provocation paradigm

|  | **Subjective Picture Block Anxiety Ratings** | | |
| --- | --- | --- | --- |
|  | **Checking** | **Washing** | **Symmetry** |
| OCI – Checking | R=0.43; p<0.006 | N/A | N/A |
| OCI – Washing | N/A | R=0.73; p<0.001 | N/A |
| OCI – Symmetry | N/A | N/A | R=0.60; p<0.001 |

**Neuroimaging Regressions with OCI-R subscale scores**

Supplementary analyses were conducted in the patient group to assess the associations between OCI washing, symmetry and checking subscale scores with their corresponding symptom dimension contrasts. Specifically, OCI subscale scores were used as covariates of interest while controlling for ADHD presence and medication usage in the patient group. There were no significant associations between OCI scores and neural activity for any of the dimensional contrasts when correcting for multiple comparisons (i.e. cluster defining threshold of p<0.001 uncorrected, FWE cluster size correction of p<0.05).


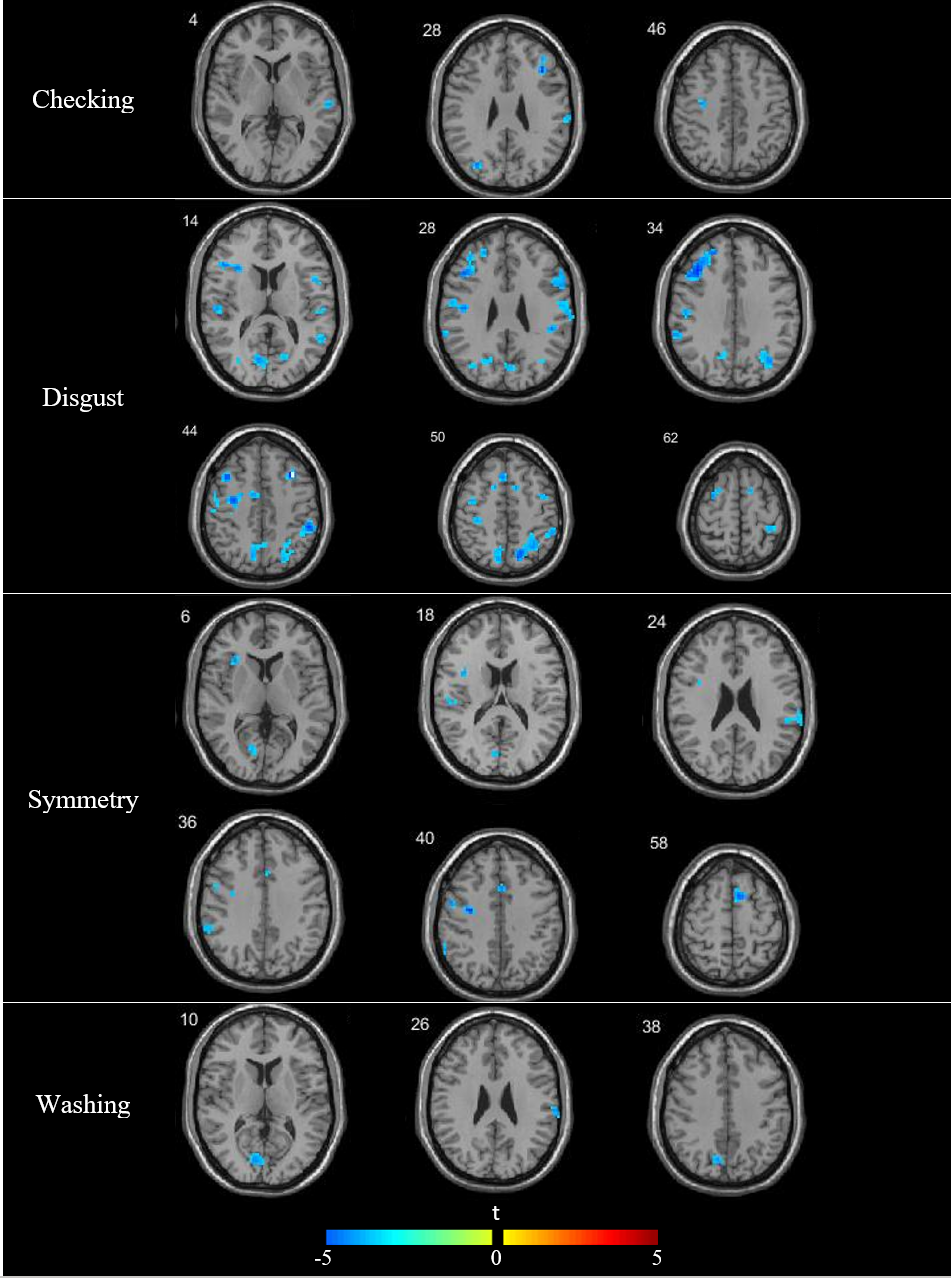


Supplementary Figure 1. Within-patient group regressions with Y-BOCS scores for each of the provocation conditions


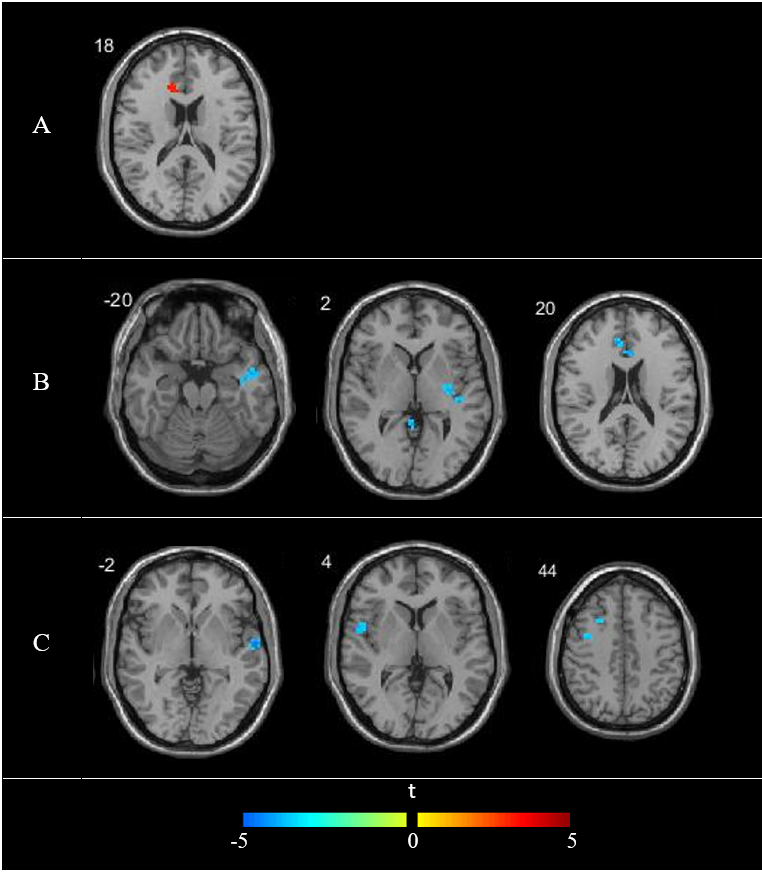


Supplementary Figure 2. A) Within-patient group regression with YGTSS total tic scores for the symmetry condition. B) Within-patient regression with DPSS sensitivity scores for the disgust condition. C) Within-patient regression with USP-SPS scores for the disgust condition.
